# Supplementary material for: Holocene shifts in marine mammal distributions around Northern Greenland revealed by sedimentary ancient DNA
Source: Nat Commun. 2025 May 15;16:4543. doi: 10.1038/s41467-025-59731-0 (PMC12081675; doi:10.1038/s41467-025-59731-0)
Supplement: Supplementary file 1 — Supplementary Information [file 41467_2025_59731_MOESM1_ESM.pdf]

Holocene shifts in marine mammal distributions around Northern Greenland revealed by sedimentary ancient DNA

Lennart Schreiber[1,2,*], Sofia Ribeiro[1,2,*,†], Rebecca Jackson[3], Anna Bang Kvorning[1,2], Kevin Nota[4], Matt O'Regan[5], Christof Pearce[6], Frederik Seersholm[1], Marit-Solveig Seidenkrantz[6], Heike H. Zimmermann[2], Eline D. Lorenzen[1,*,†]

1 Globe Institute, University of Copenhagen, Denmark

2 Department of Glaciology and Climate, Geological Survey of Denmark and Greenland, Øster Voldgade 10, 1350 Copenhagen K, Denmark

3 MARUM – Center for Marine Environmental Sciences, Bremen, Germany

4 Department of Evolutionary Genetics, Max Planck Institute for Evolutionary Anthropology, Leipzig, Germany

5 Department of Geological Sciences, Stockholm University, Stockholm, Sweden

6 Department of Geoscience, Arctic Research Center, and iClimate Center, Aarhus University, Aarhus, Denmark

Supplementary Figures

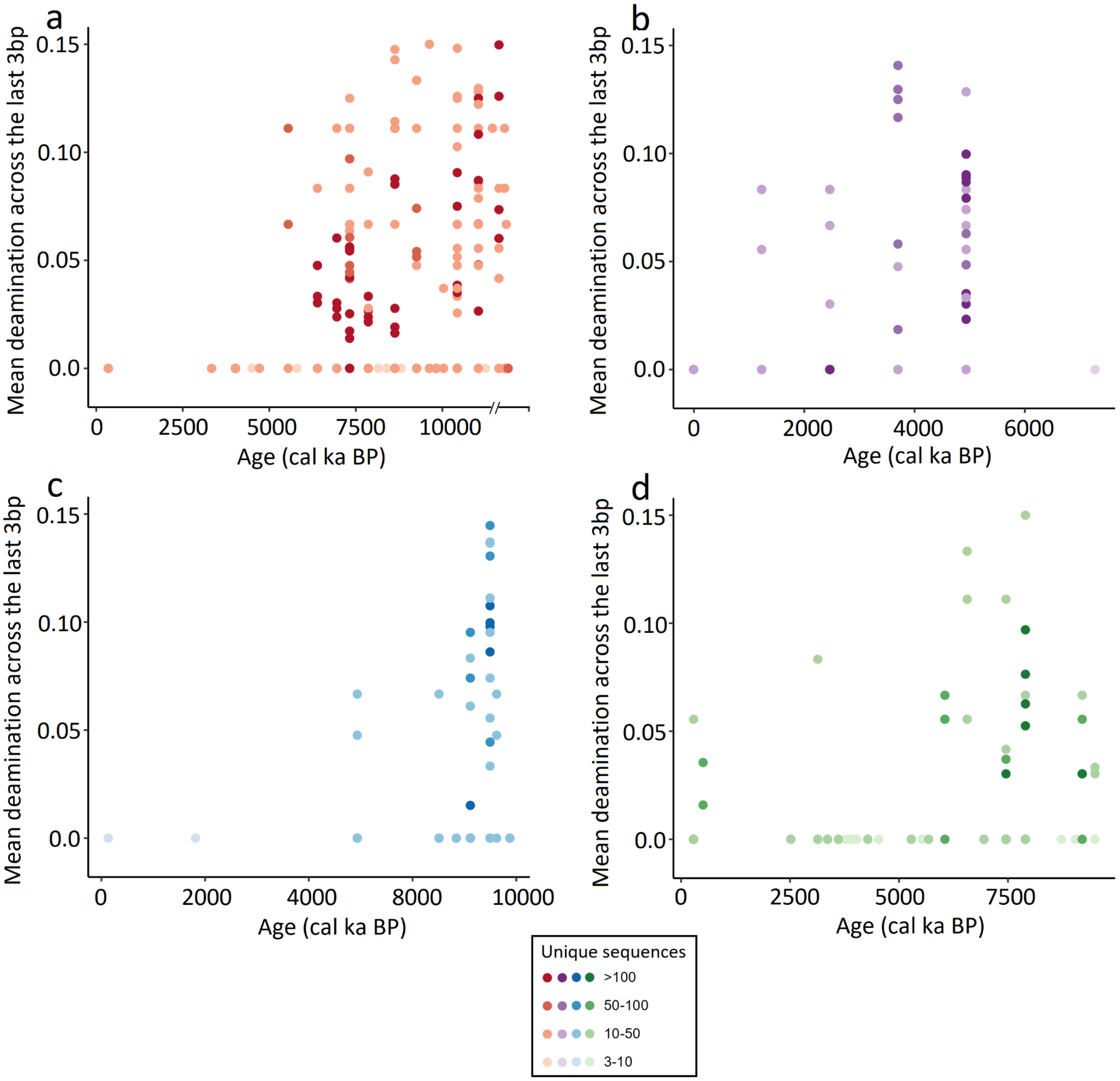

Supplementary Figure 1 DNA damage in the shotgun and hybridization capture sequencing data across the four marine sediment cores analysed. Mean nucleotide deaminations per taxon and estimated median age were calculated across the last 3bp of the forward and reverse strand. **a** Melville Bay 26G. **b** Hall Basin 24PC. **c** Lincoln Sea 12-GC. **d** North-East Greenland 73G. Colors indicate the number of unique sequences the nucleotide deamination calculations were based on.

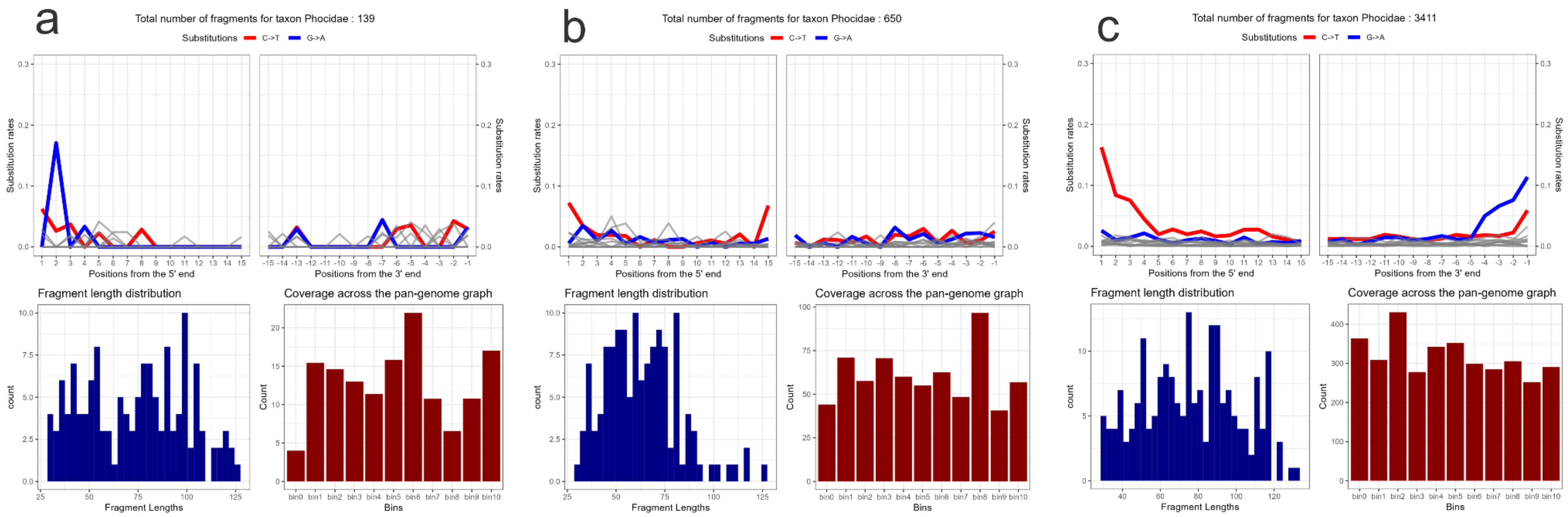

Supplementary Figure 2 Deamination profiles, fragment length distribution and coverage of marine sediement core Melville Bay 26G DNA sequences assigned to Phocidae. Duplicate removed DNA sequences were binned by sample ages and processed using *euka*[1]. Samples with estimated ages 0 - 3 cal ka BP did not contain sufficient sequences assigned to Phocidae. **a** Samples with estimated ages 3 - 6 cal ka BP. **b** Samples with estimated ages 6 - 9 cal ka BP. **c** Samples with estimated ages 9 - 12 cal ka BP.

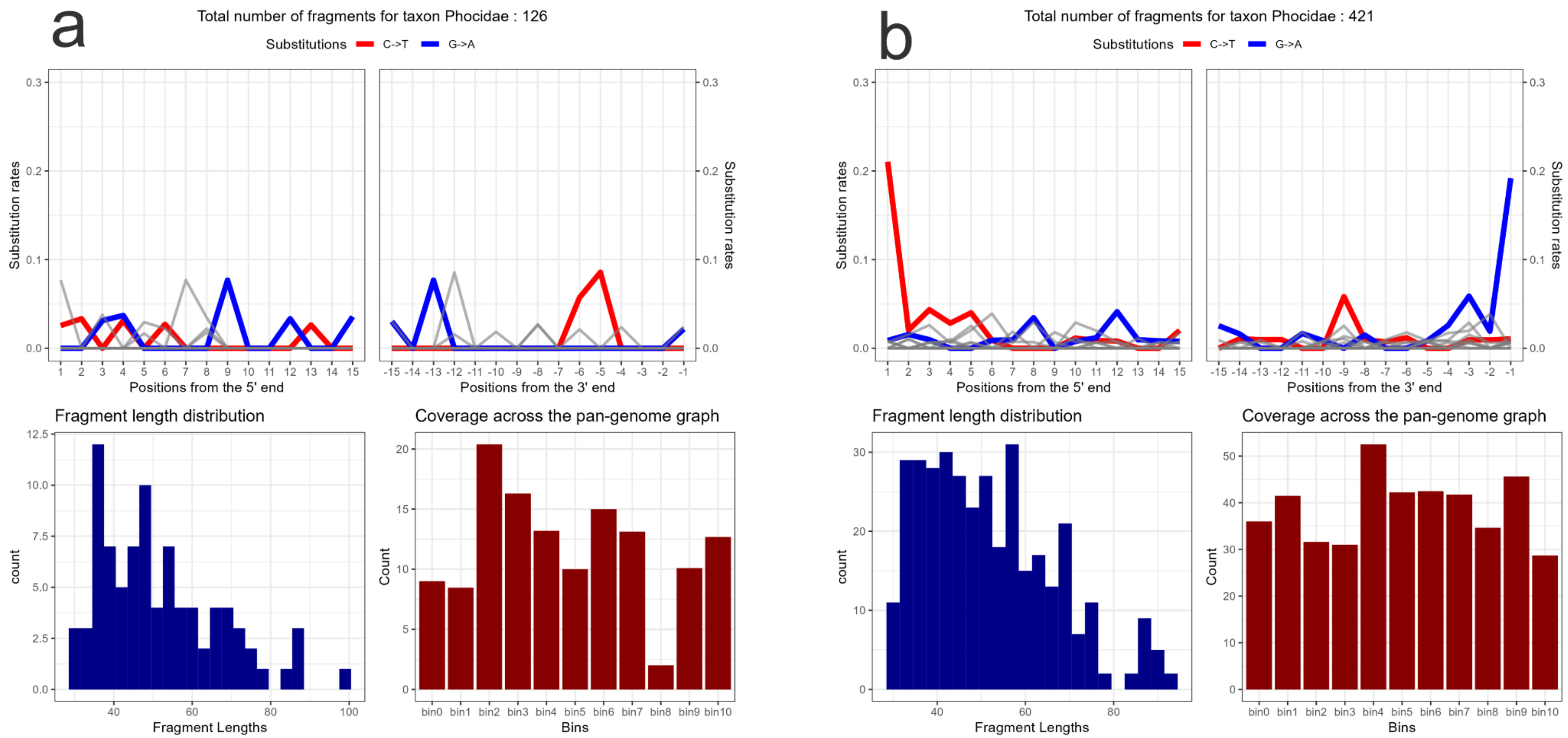

Supplementary Figure 3 Deamination profiles, fragment length distribution and coverage of marine sediment core Hall Basin 24PC DNA sequences assigned to Phocidae. Duplicate removed DNA sequences were binned by sample ages and processed using *euka*[1]. **a** Samples with estimated ages 0 - 3 cal ka BP. **b** Samples with estimated ages 3 - 6 cal ka BP. Samples with estimated ages >6 cal ka BP did not contain sufficient sequences assigned to Phocidae.

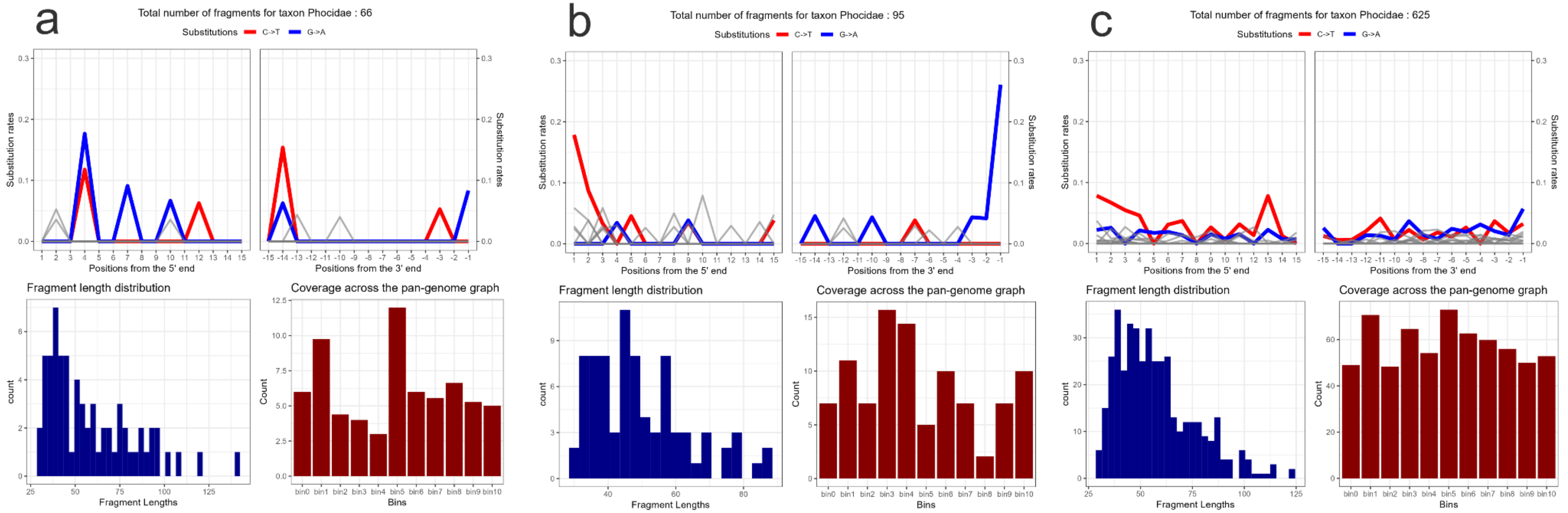

Supplementary Figure 4 Deamination profiles, fragment length distribution and coverage of marine sediment core Lincoln Sea 12-GC DNA sequences assigned to Phocidae. Duplicate removed DNA sequences were binned by sample ages and processed using *euka* [1]. **a** Samples with estimated ages 0 - 3 cal ka BP. **b** Samples with ages 3 - 6 cal ka BP. **c** Samples with estimated ages 6 - 9 cal ka BP. Samples with estimated ages >9 cal ka BP did not contain sufficient sequences assigned to Phocidae.

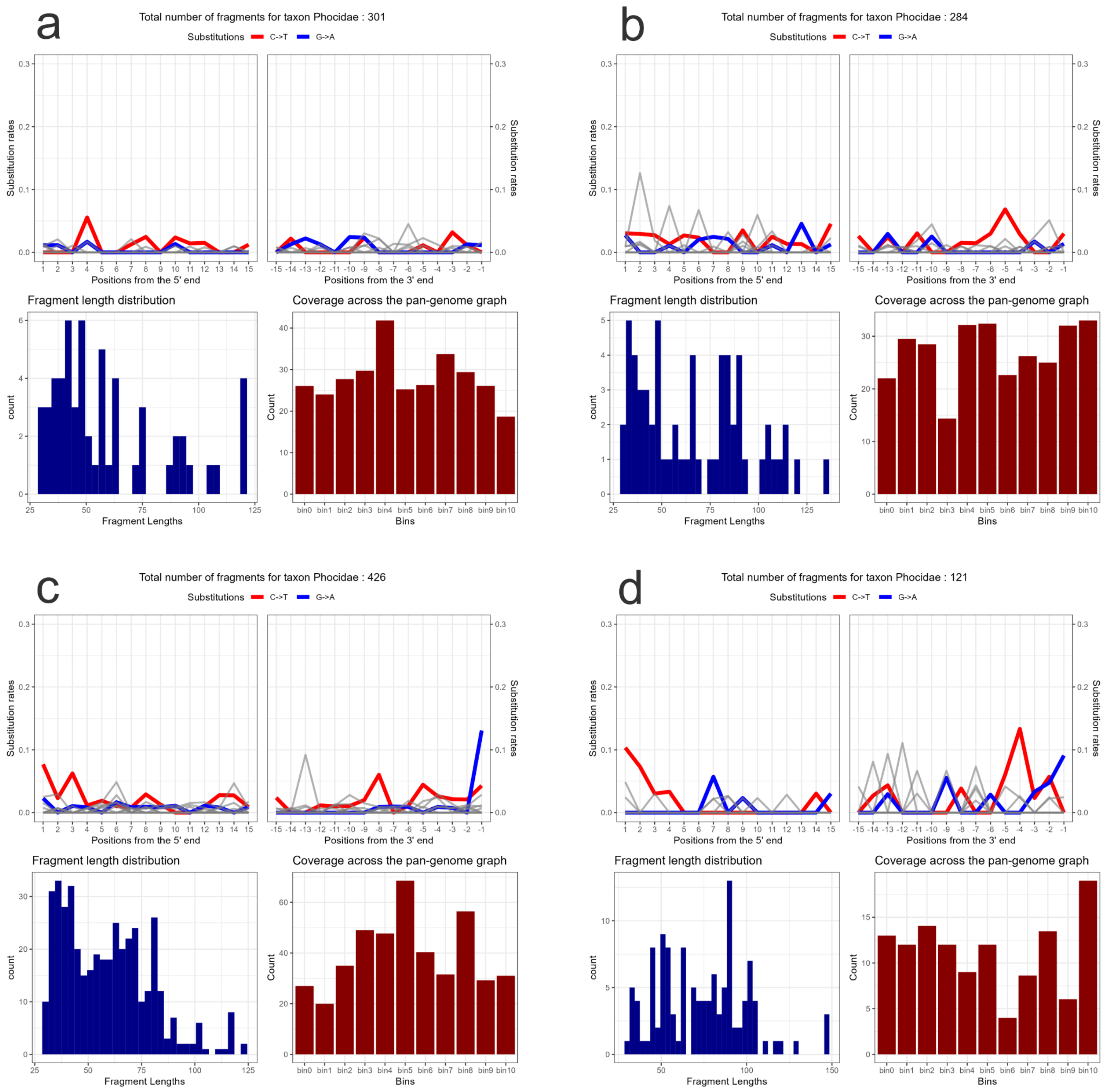
a
Total number of fragments for taxon Phocidae : 301
Substitutions C->T G->A
Substitution rates
Positions from the 5' end
Positions from the 3' end
Fragment length distribution
count
Fragment Lengths
Coverage across the pan-genome graph
Count
Bins
b
Total number of fragments for taxon Phocidae : 284
Substitutions C->T G->A
Substitution rates
Positions from the 5' end
Positions from the 3' end
Fragment length distribution
count
Fragment Lengths
Coverage across the pan-genome graph
Count
Bins
c
Total number of fragments for taxon Phocidae : 426
Substitutions C->T G->A
Substitution rates
Positions from the 5' end
Positions from the 3' end
Fragment length distribution
count
Fragment Lengths
Coverage across the pan-genome graph
Count
Bins
d
Total number of fragments for taxon Phocidae : 121
Substitutions C->T G->A
Substitution rates
Positions from the 5' end
Positions from the 3' end
Fragment length distribution
count
Fragment Lengths
Coverage across the pan-genome graph
Count
Bins

Supplementary Figure 5 Deamination profiles, fragment length distribution and coverage of marine sediment core North-East Greenland 73G DNA sequences assigned to Phocidae. Duplicate removed DNA sequences were binned by sample ages and processed using *euka*[1]. **a** Samples with estimated ages 0 - 3 cal ka BP. **b** Samples with estimated ages 3 - 6 cal ka BP. **c** Samples with estimated ages 6 - 9 cal ka BP. **d** Samples with estimated ages 9 - 10 cal ka BP.

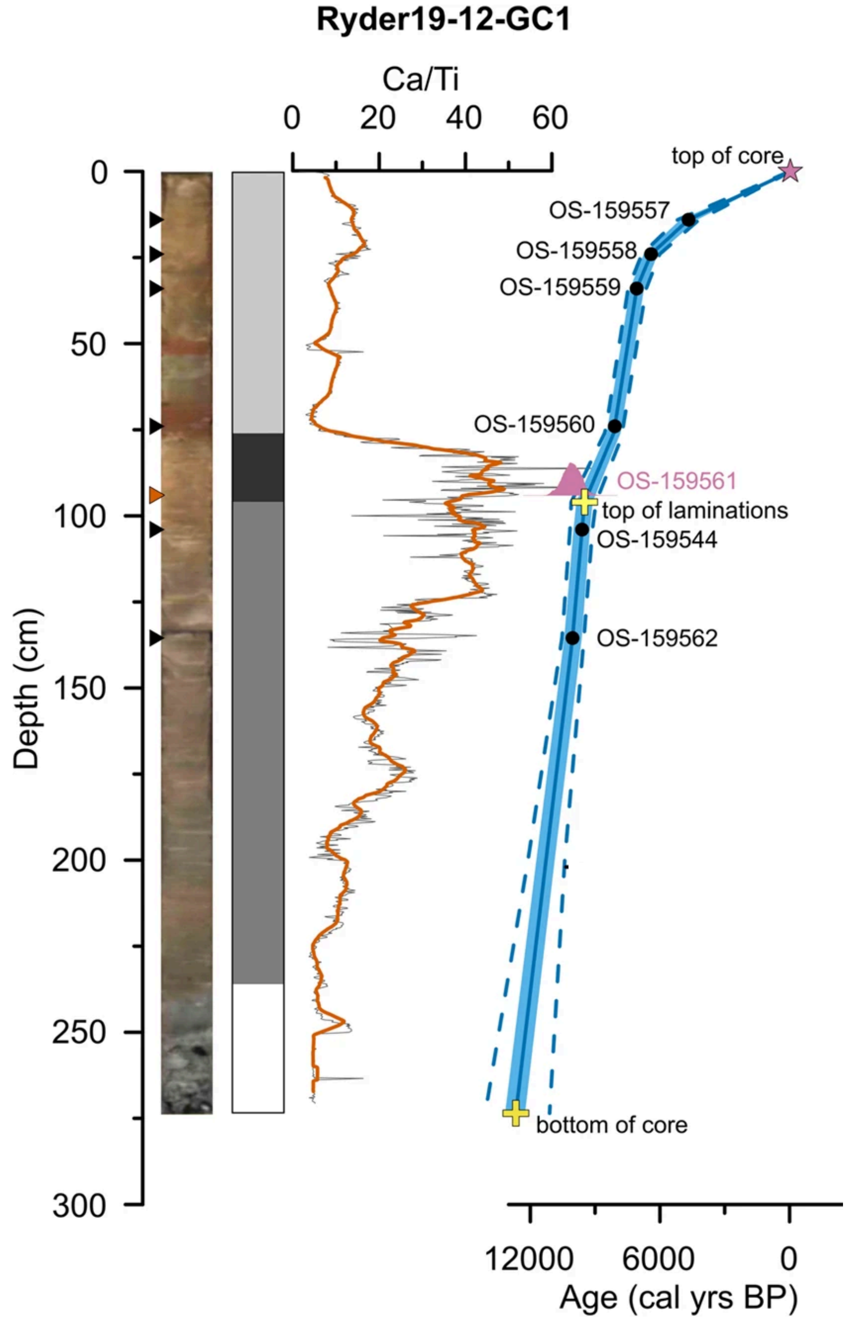

Supplementary Figure 6 Age-depth model of marine sediment core Lincoln Sea 12-GC (otherwise known as Ryder19-12-GC1). Reproduced from Detlef et al.[2] with permission from the publisher under the Creative Commons Attribution 4.0 International License (creativecommons.org/licenses/by/4.0/).

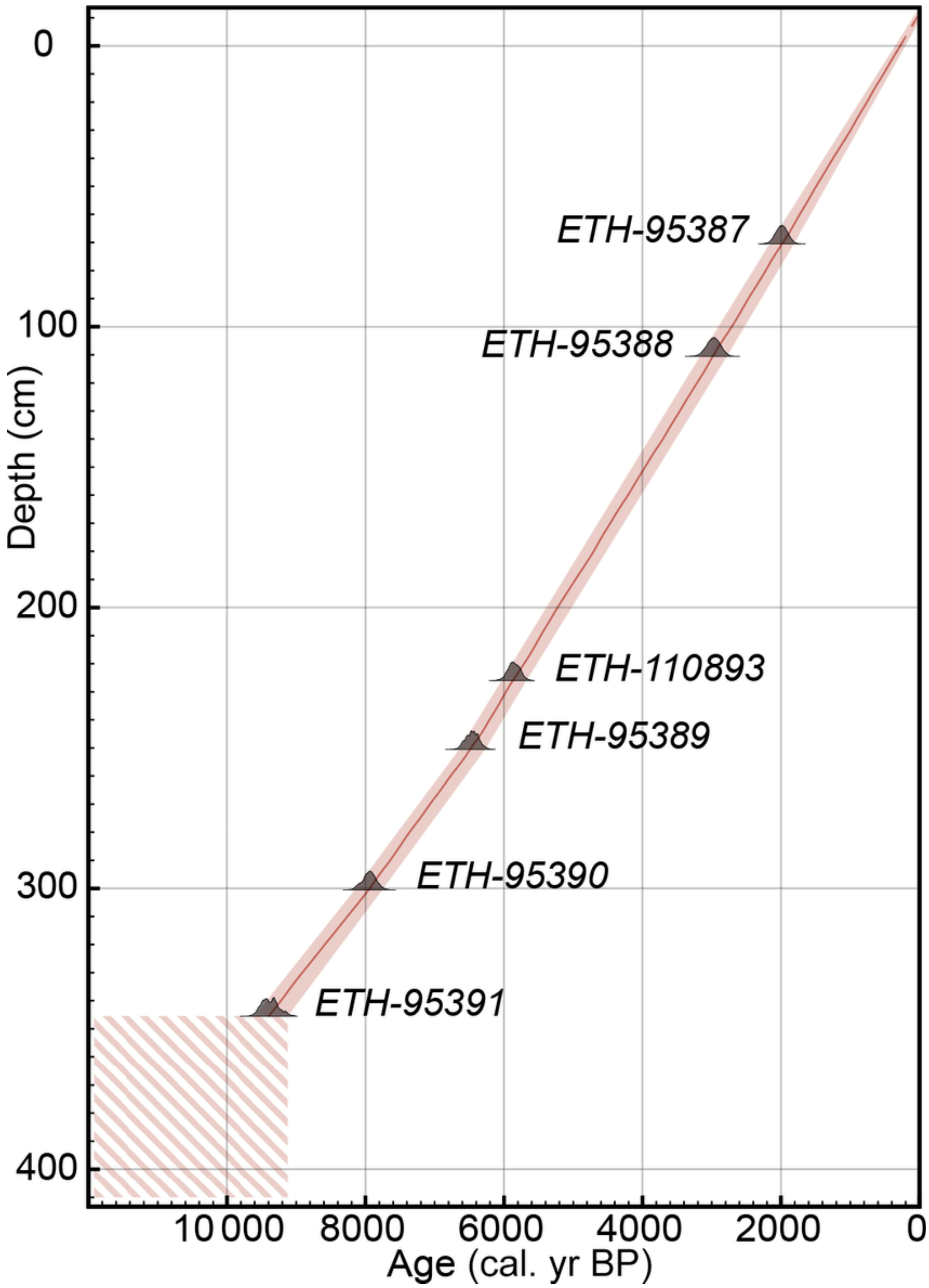

Supplementary Figure 7 Age-depth model of marine sediment core North-East Greenland 73G (otherwise known as DA17-NG-ST07-073G). Reproduced from Pados-Dibattista et al.[3] with permission from the publisher under the Creative Commons Attribution 4.0 International License (creativecommons.org/licenses/by/4.0/).

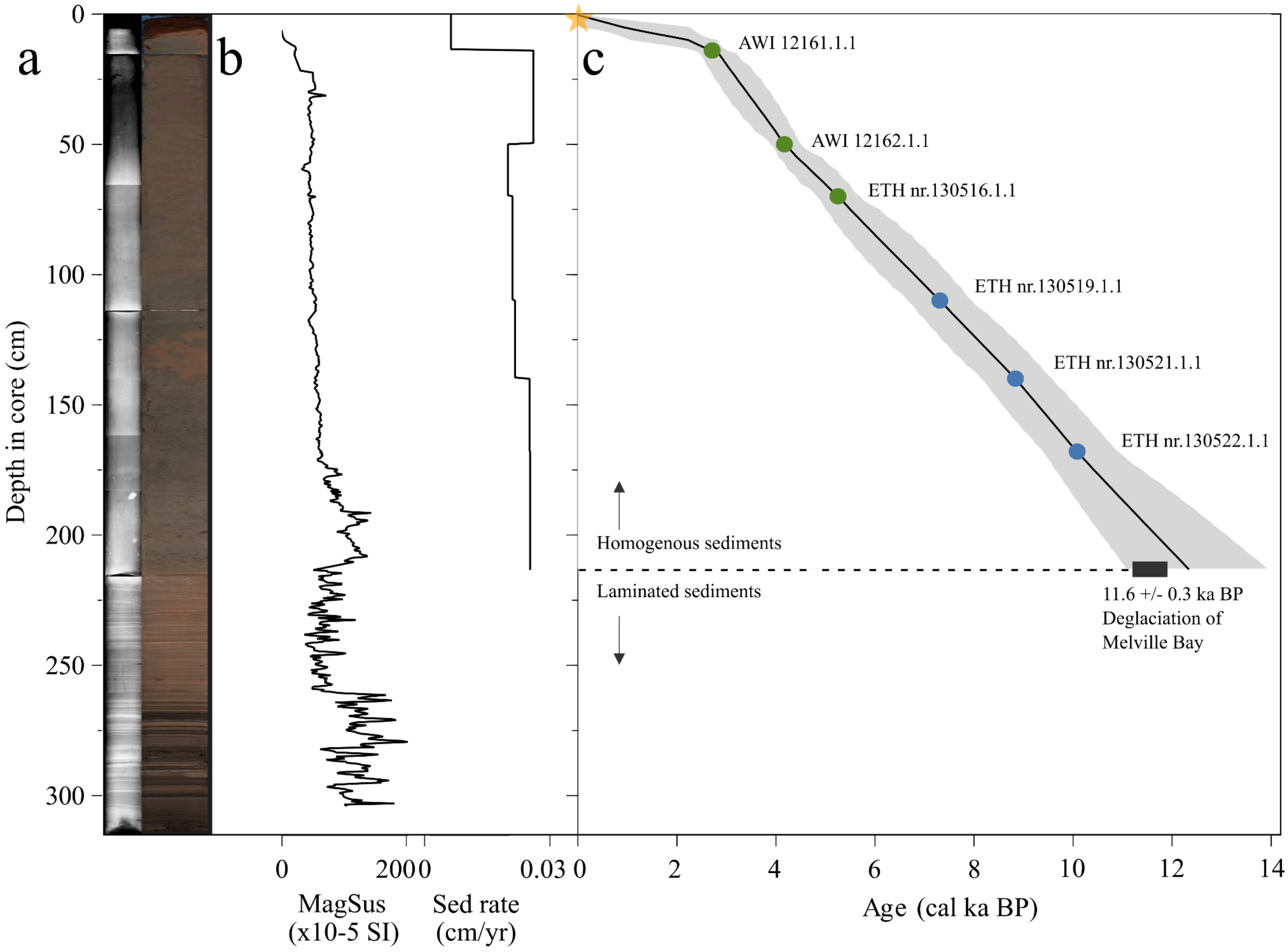

Supplementary Figure 8 Chronology of marine sediment core Melville Bay 26G (otherwise known as LK21-IC-st26-GC1). a X-ray and high resolution optical line scan images. **b** Magnetic susceptibility (MagSus, x10^{-6} SI) and sedimentation rate (cm yr^{-1}). **c** The age-depth model with green dots displaying ^{14}C dates of three mixed planktonic foraminifera samples, blue dots displaying ^{14}C dates of three mixed benthic foraminifera samples, and the gray envelope displaying the age-depth uncertainty range for the derived age model. The yellow star indicates an age constraint (as discussed in the Methods section) and the timing of the deglaciation of Melville Bay (estimated 11.6 ± 0.3 ka BP[4]) is included for reference.

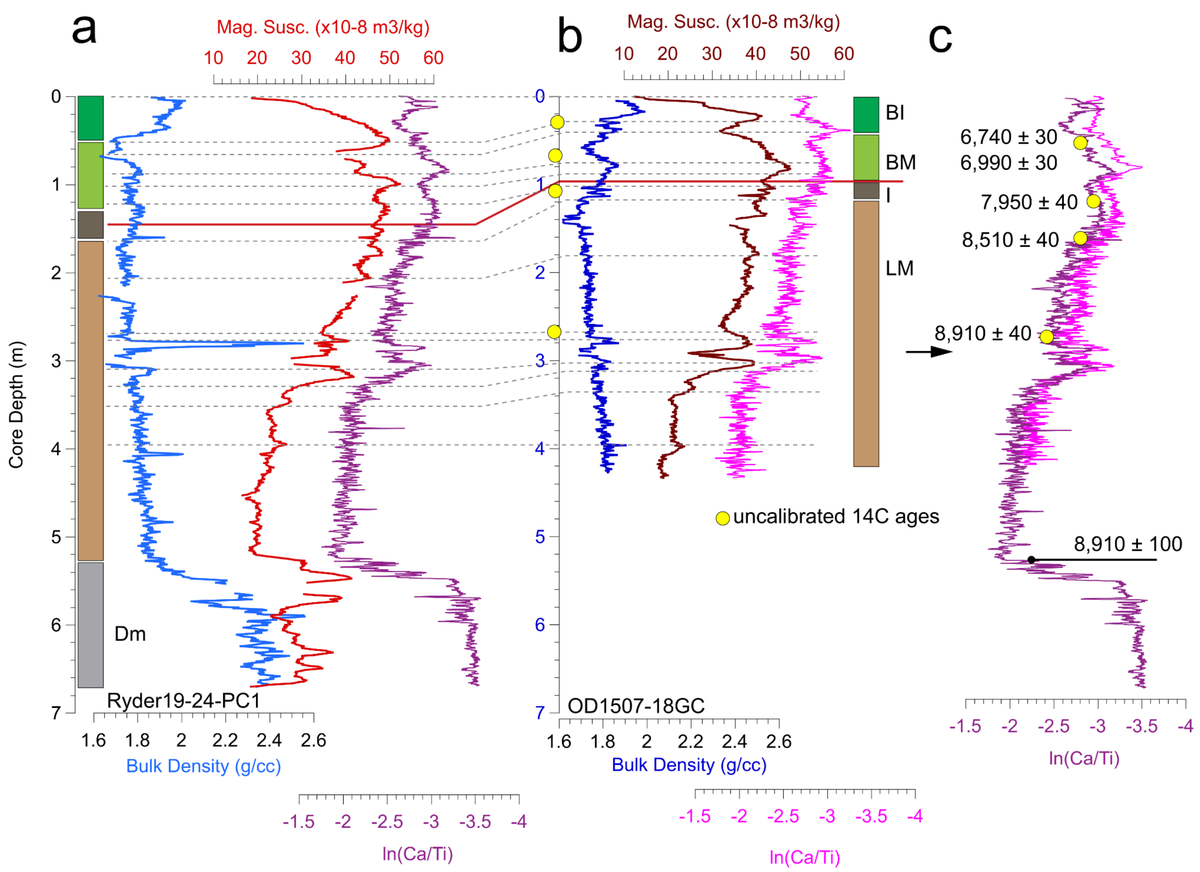

Supplementary Figure 9 Age-depth derivation of marine sediment core Hall Basin 24PC (otherwise known as Ryder19-24-PC1). a Bulk Density, Magnetic Susceptibility and XRF-scanning data of sediment core Ryder19-24PC. **b** Bulk Density, Magnetic Susceptibility and XRF-scanning data of sediment core OD1507-18GC (collected at the same station as Ryder19-24PC[5]). Reproduced from Jennings et al.[5] with permission from the publisher under the Creative Commons Attribution 4.0 International License (creativecommons.org/licenses/by/4.0/). **c** The common age-depth scale based on correlating the XRF-scanning data of the two sediment cores (as discussed in the Methods section).

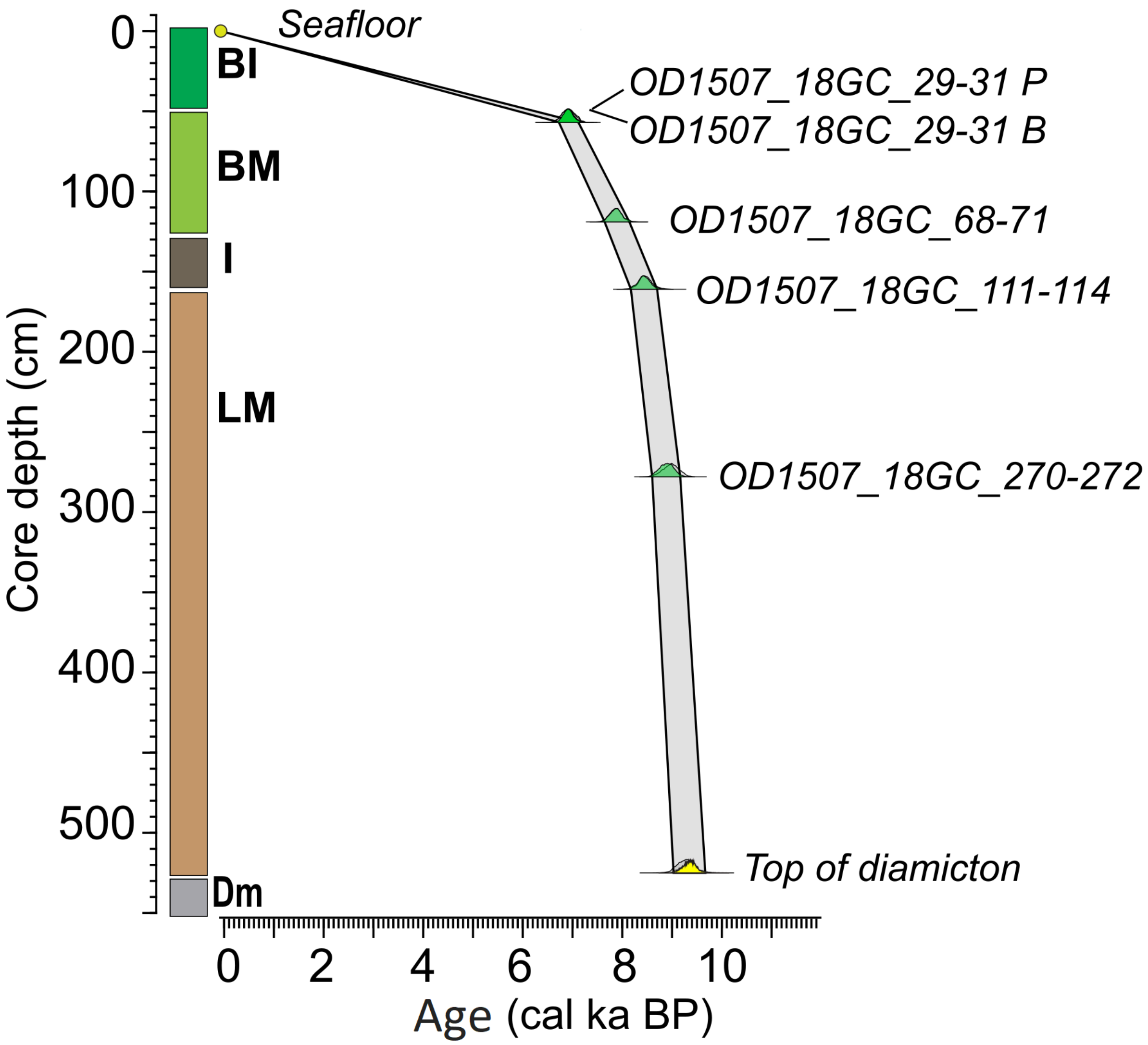

Supplementary Figure 10 Age-depth model of marine sediment core Hall Basin 24PC (otherwise known as Ryder19-24-PC1). The age-depth model is based on a derivation of radiocarbon dates from the marine sediment core OD1507-18GC[5] (Supplementary Figure 9).

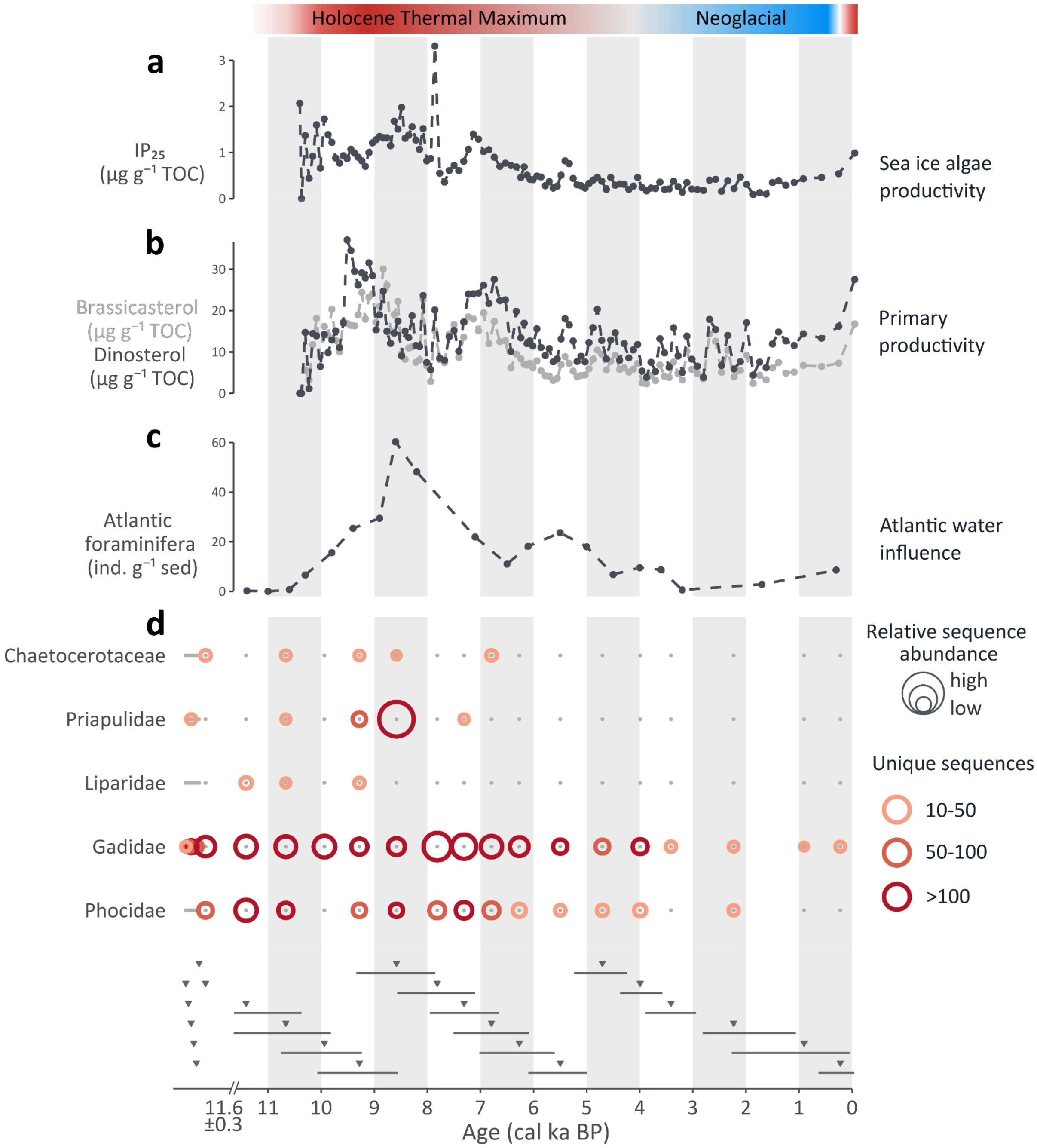

Supplementary Figure 11 Paleoceanographic reconstructions and DNA detections for marine sediment core Melville Bay 26G. a Sea-ice biomarker IP_{25} and **b** primary-productivity biomarkers brassicasterol and dinosterol are derived from marine sediment core GeoB19927-3[6]. **c** High numbers of benthic foraminifera with preferred habitat of Atlantic-sourced waters indicate the influence of chilled Atlantic water, points represent counts per gram sediment (this study). **d** Eukaryote detections through shotgun sequencing. Triangles show the estimated median age of each sample, with horizontal lines indicating confidence intervals. Samples with estimated median ages >11.6 cal ka BP likely represent a short time period at or just prior to the deglaciation of Melville Bay (estimated 11.6 ± 0.3 ka BP[4]).

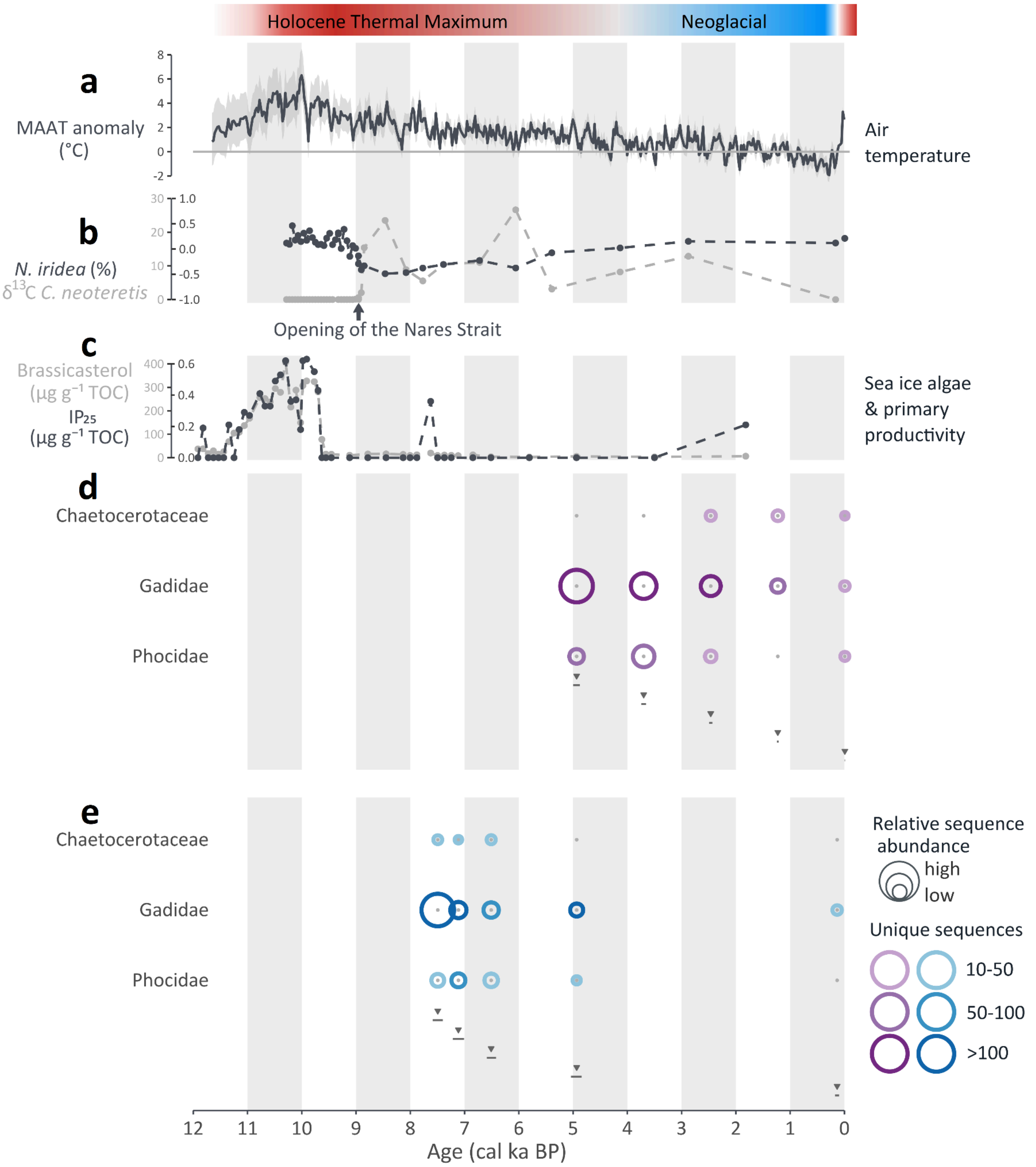

Supplementary Figure 12 Palaeoenvironmental reconstructions and DNA detections for marine sediment cores Hall Basin 24PC and Lincoln Sea 12-GC. a Temperature reconstruction based on the oxygen isotopic signature ($\delta^{18}O$) at the Agassiz ice cap as compared to the pre-industrial (1850-1900 CE) average[2,7]. **b** Evidence for the opening of Nares Strait (derived from core HLY03-01-05GC[8]). **c** Sea-ice biomarker IP_{25} and primary-productivity biomarker brassicasterol, derived from Lincoln Sea 12-GC[2]. **d** Eukaryote detections through shotgun sequencing for Hall Basin 24PC. **e** Eukaryote detections through shotgun sequencing for Lincoln Sea 12-GC. Triangles show the estimated median age of each DNA sample, with horizontal lines indicating confidence intervals.

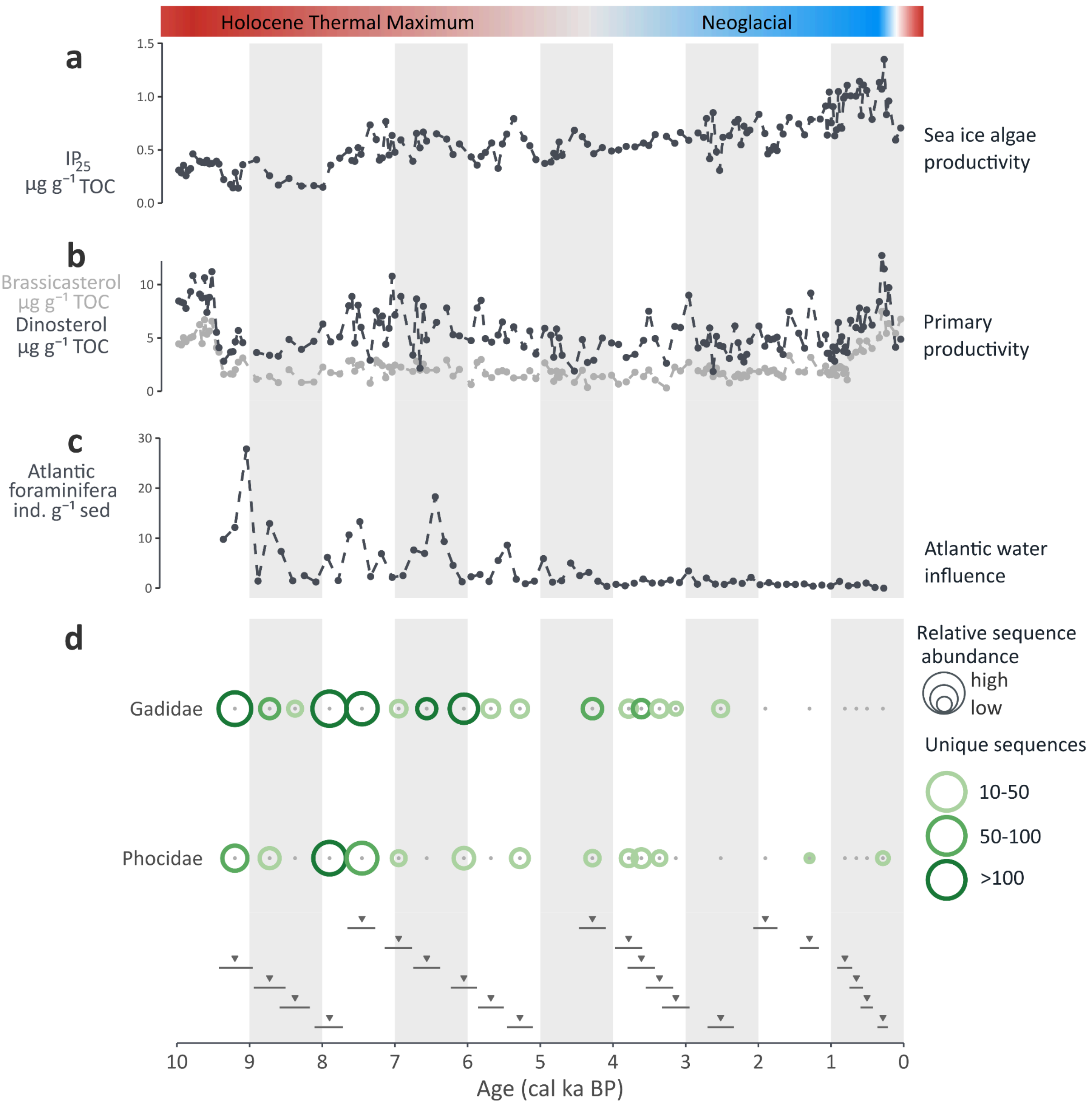

Supplementary Figure 13 Paleoceanographic reconstructions and DNA detections for marine sediment core North-East Greenland 73G. a Sea-ice biomarker IP_{25} and **b** primary productivity biomarkers brassicasterol and dinosterol are derived from marine sediment core PS93/025-2[9]. **c** High numbers of benthic foraminifera with preferred habitat of Atlantic-sourced waters indicate the influence of Atlantic water, points represent counts per gram sediment (derived from marine sediment core North-East Greenland 73G[3]. **d** Eukaryote detections through shotgun sequencing. Triangles show the estimated median age of each DNA sample, with horizontal lines indicating confidence intervals.

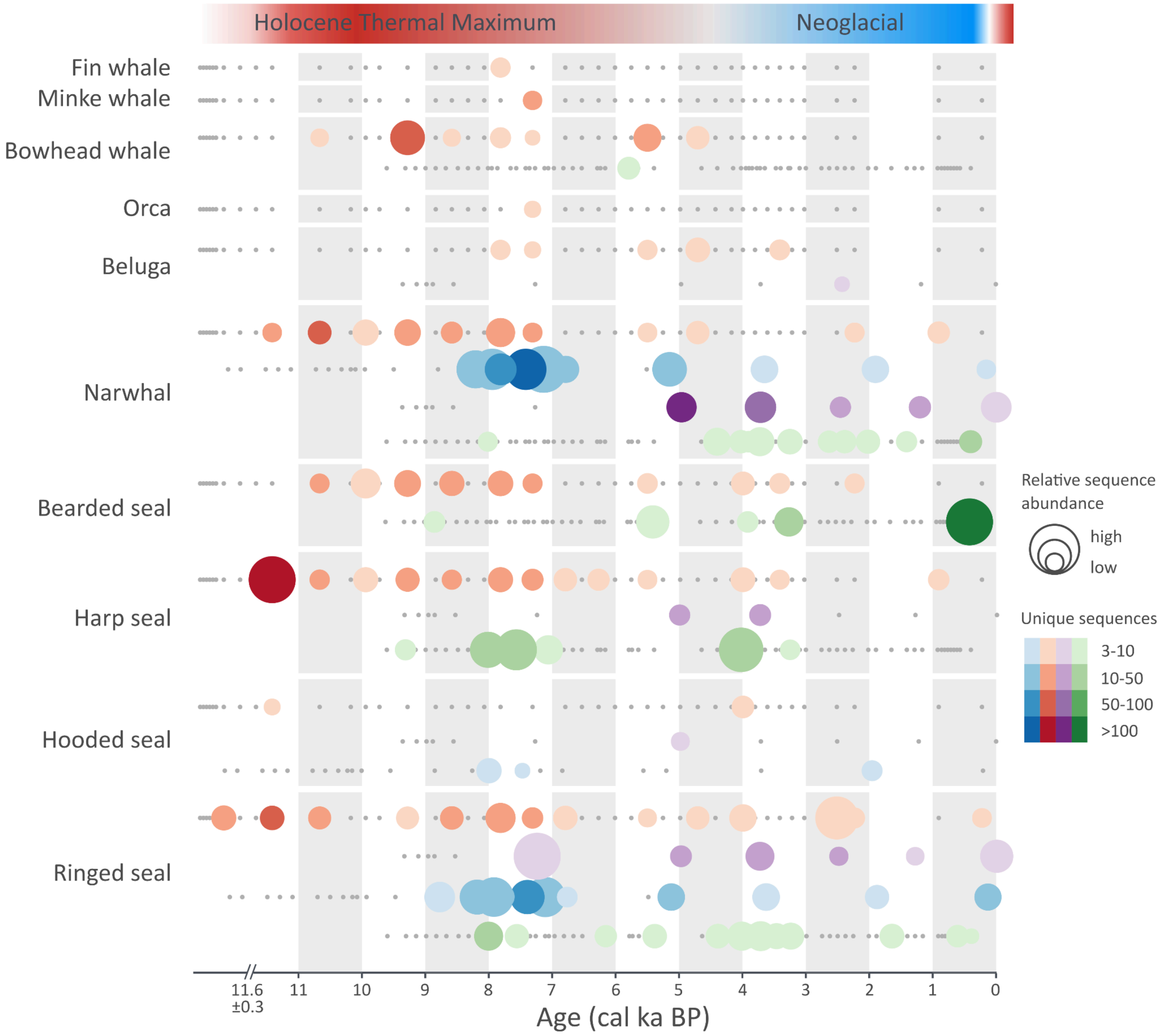

Supplementary Figure 14 Marine mammal detections through hybridization capture across the four marine sediment cores. The detections are grouped by species. The four colours correspond to each of the four cores: Melville Bay 26G (red; 75° N; LK21-IC-st26-GC1); Hall Basin 24PC (purple; 82° N; Ryder19-24-PC1); Lincoln Sea 12-GC (blue; 83° N; Ryder19-12-GC1); North-East Greenland 73G (green; 79° N; DA17-NG-ST07-073G).

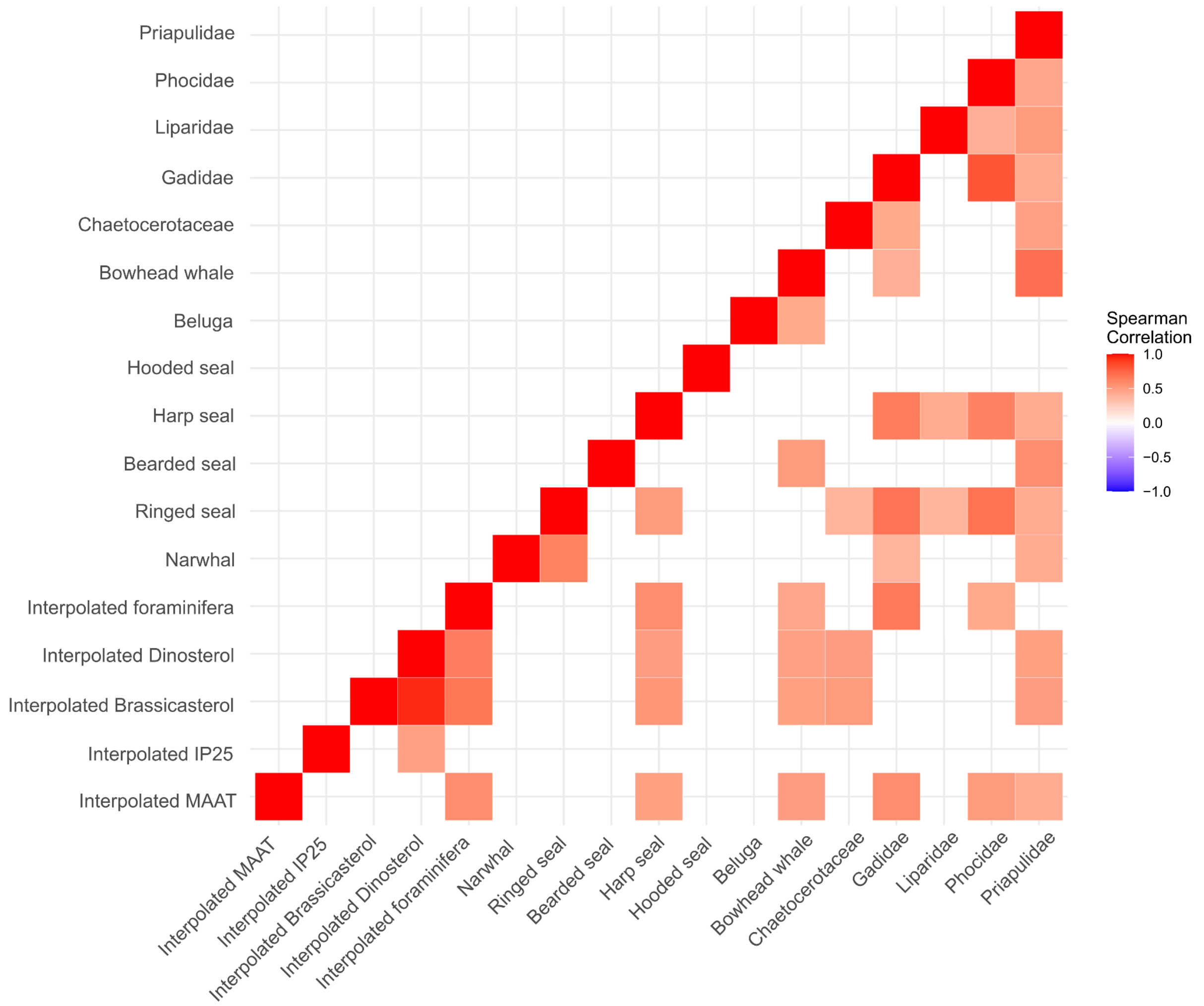

Supplementary Figure 15 Correlation matrix of DNA detections and interpolated paleoenvironmental proxies. For each sample and across sediment cores, DNA detections based on shotgun sequencing and hybridisation capture and linearly interpolated paleoenvironmental proxy measurements were compiled in a matrix. Pairwise Spearman's rank correlation coefficients were then calculated and filtered for significant ($p < 0.05$) correlations (Supplementary Data 7).

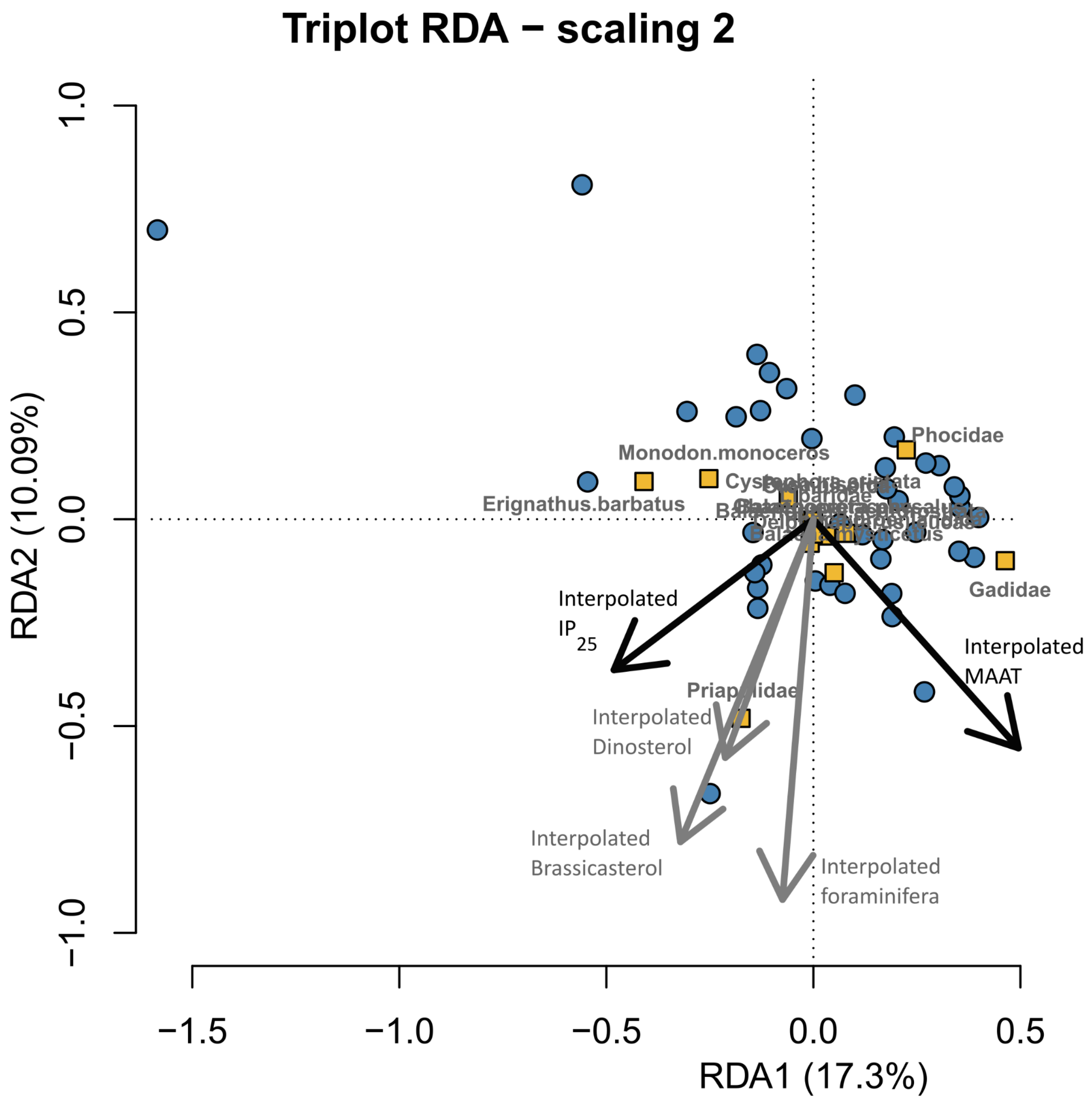

Supplementary Figure 16 Redundancy analysis of shotgun sequencing and hybridisation capture detections for the four marine sediment cores. For each sample and across the four cores Melville Bay 26G, Hall Basin 24PC, Lincoln Sea 12-GC, and North-East Greenland 73G, species-level detections based on hybridisation capture, family-level detections based on shotgun sequencing, and linearly interpolated paleoenvironmental proxy measurements were compiled in a matrix. A redundancy analysis (RDA) was performed followed by an ANOVA-like permutation test to identify variables that contributed significantly to explaining the diversity of the samples. The interpolated air temperature (MAAT[7]) and sea-ice biomarker IP_{25}[6,9] proved to be significant (Pr(>F)<0.05; black arrow and font), and all other (insignificant) variables are indicated by gray color.

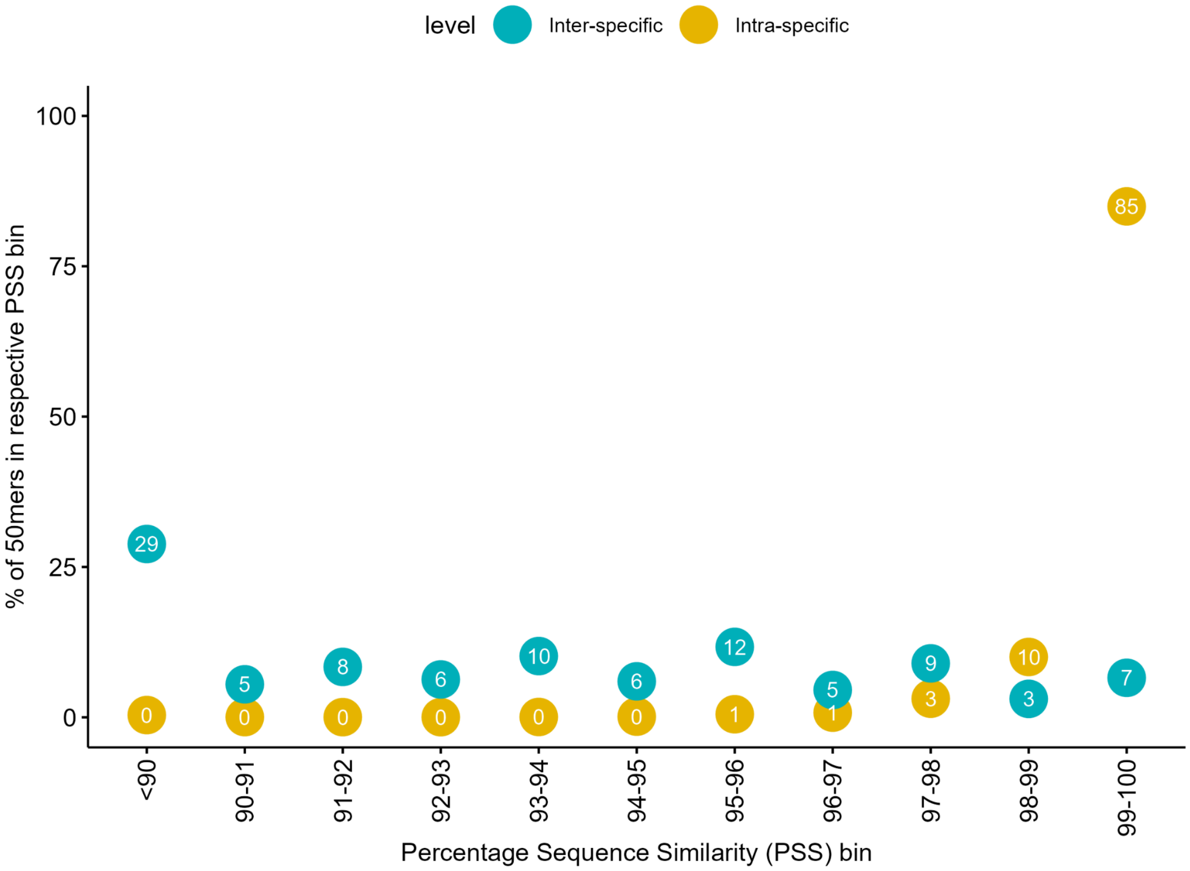

Supplementary Figure 17 Pairwise sequence similarity calculations. Inter-specific pairwise sequence similarities (in blue) were calculated for mitochondrial reference genomes belonging to the target group of Arctic marine mammals and their closest relatives (i.e. other species belonging to the subfamilies Phocinae, Balaenidae, and Monodontidae). Intra-specific pairwise sequence similarities (in yellow) were calculated individually for each target species (e.g. all available mitochondrial reference genomes of bowhead whales, narwhals, etc.).

Supplementary references

1. Vogel, N. A. *et al.* euka: Robust tetrapodic and arthropodic taxa detection from modern and ancient environmental DNA using pangenomic reference graphs. *Methods Ecol. Evol.* **14**, 2717–2727 (2023).

2. Detlef, H. *et al.* Seasonal sea-ice in the Arctic's last ice area during the Early Holocene. *Commun. Earth Environ.* **4**, 86 (2023).

3. Pados-Dibattista, T., Pearce, C., Detlef, H., Bendtsen, J. & Seidenkrantz, M.-S. Holocene palaeoceanography of the Northeast Greenland shelf. *Clim. Past* **18**, 103–127 (2022).

4. Søndergaard, A. S. *et al.* Early Holocene collapse of marine-based ice in northwest Greenland triggered by atmospheric warming. *Quat. Sci. Rev.* **239**, 106360 (2020).

5. Jennings, A. *et al.* Modern and early Holocene ice shelf sediment facies from Petermann Fjord and northern Nares Strait, northwest Greenland. *Quat. Sci. Rev.* **283**, 107460 (2022).

6. Saini, J. *et al.* Holocene variability in sea ice and primary productivity in the northeastern Baffin Bay. *arktos* **6**, 55–73 (2020).

7. Lecavalier, B. S. *et al.* High Arctic Holocene temperature record from the Agassiz ice cap and Greenland ice sheet evolution. *Proc. Natl. Acad. Sci.* **114**, 5952–5957 (2017).

8. Jennings, A. *et al.* The Holocene History of Nares Strait: Transition from Glacial Bay to Arctic-Atlantic Throughflow. *Oceanography* **24**, 26–41 (2011).

9. Syring, N. *et al.* Holocene changes in sea-ice cover and polynya formation along the eastern North Greenland shelf: New insights from biomarker records. *Quat. Sci. Rev.* **231**, 106173 (2020).
