# Supplementary material for: Holocene shifts in marine mammal distributions around Northern Greenland revealed by sedimentary ancient DNA
Source: Nat Commun. 2025 May 15;16:4543. doi: 10.1038/s41467-025-59731-0 (PMC12081675; doi:10.1038/s41467-025-59731-0)
Supplement: Supplementary file 2 — Description of Addtional Supplementary Files [file 41467_2025_59731_MOESM2_ESM.pdf]

Supplementary Data 1 Shotgun sequencing data. For DNA assigned to eukaryotes (green columns), all unique sequences on a taxonomic family level with a minimum of 10 unique sequences are shown per sample and for DNA assigned to prokaryotes (red columns), all unique sequences on a taxonomic phylum level with a minimum of 10 unique sequences are shown. For a detailed description of the bioinformatic processing of raw sequencing data, please refer to the Methods section.

Supplementary Data 2 Age dates of marine sediment core Melville Bay 26G (LK21-IC-st26-GC1). Radiocarbon dates were calibrated in R with the package rbacon (Blaauw et al., 2024) using Marine20 (Heaton et al., 2020) and a local reservoir offset (ΔR) of -49 ± 59 years (Pearce et al., 2023).

Supplementary Data 3 Detectable 210Pb measurements in the marine sediment core Melville Bay 26G (LK21-IC-st26-GC1). The upper 10 cm of the sediment core were tested for 210Pb excess activity using a Canberra ultralow-background Ge-detector and the Constant Rate of Supply (CRS) model (Andersen, 2017).

Supplementary Data 4 Details on the age-depth derivation of Hall Basin 24PC (Ryder19-24-PC1). Five radiocarbon dates from the adjacent marine sediment core OD1507-18GC (450 m away; Jennings et al., 2022) were migrated onto the depth scale of Hall Basin 24PC after a common depth scale was constructed based on bulk density, magnetic susceptibility, and XRF-scanning (Fig. S5).

Supplementary Data 5 Known spatial distribution of the detected marine mammal species. Bold font indicates year-round presence in the area; regular font indicates summer presence of the species. Question marks (?) indicate the expected presence of the species in the area, despite the absence of observational data, based on personal communication with experts from the Greenland Institute of Natural Resources (M.P. Heide-Jørgensen, 19th Jan. 2024 and A. Rosing-Asvid, 10th Jan. 2024).

Supplementary Data 6 Selected foraminifera counts of marine sediment core Melville Bay 26G (LK21st.26GC). Only benthic foraminifera (calcareous and agglutinated) foraminifera associated with chilled Atlantic Water masses are shown.

Supplementary Data 7 Details of Spearman's rank correlation coefficient analysis. The correlation coefficients of DNA detections based on shotgun sequencing and hybridisation capture and linearly interpolated paleoenvironmental proxy measurements were filtered for significant ($p < 0.05$) correlations.

Supplementary Data 8 Age dates of marine mammal fossils discussed in the main text. Radiocarbon ages were retrieved from the literature. Dates were calibrated in R with the package

rbacon (Blaauw et al., 2024), using appropriate reservoir offsets based on the geographic location of fossil samples, and the Marine20 calibration curve (Heaton et al., 2020).

Supplementary Data 9 Mitochondrial hybridization capture panel. The mitochondrial genomes of each species used to design the hybridization capture panel are indicated by the NCBI accession IDs. Seven of the species included in the capture panel are endemic to the Arctic and five are temperate species typically found at lower latitudes.

Supplementary Data 10 Reproducibility of hybridization capture sequencing data. Detections are grouped by sample ID, which corresponds to samples of a specific depth in the respective sediment core. Each capture ID represents sequencing data of a specific capture experiment. Marine mammal detections overlapping between capture experiments of the same sample ID are colored in green, non-overlapping detections with less than 3 unique sequences (which was used as a cut-off for the visual presentation of the results) are colored in yellow, and non-overlapping detections with at least 3 unique sequences are colored in red. For a detailed description of the bioinformatic processing of raw sequencing data, please refer to the Methods section.

Supplementary Data 11 Hybridization capture sequencing data. Detections are grouped into marine mammals (green columns), presumed bycatch (yellow columns) and presumed contamination (red columns). Only taxonomic assignments on a species level with a minimum of 3 unique sequences are shown. For a detailed description of the bioinformatic processing of raw sequencing data, please refer to the Methods section.

Supplementary Data 12 Metadata summary of sequencing experiments. This table provides detailed metadata for the metagenomic shotgun sequencing and hybridization capture experiments, including experiment, study and sample accession numbers. The total data size (in megabases) and bases are listed alongside library-specific information, including library names, strategies, sources, and selection methods.
