# Supplementary material for: Holocene shifts in marine mammal distributions around Northern Greenland revealed by sedimentary ancient DNA
Source: Nat Commun. 2025 May 15;16:4543. doi: 10.1038/s41467-025-59731-0 (PMC12081675; doi:10.1038/s41467-025-59731-0)
Supplement: Supplementary file 4 — Reporting Summary [file 41467_2025_59731_MOESM4_ESM.pdf]

Corresponding author(s): Lennart Schreiber

Last updated by author(s): Apr 10, 2025

Reporting Summary

Nature Portfolio wishes to improve the reproducibility of the work that we publish. This form provides structure for consistency and transparency in reporting. For further information on Nature Portfolio policies, see our Editorial Policies and the Editorial Policy Checklist.

Statistics

For all statistical analyses, confirm that the following items are present in the figure legend, table legend, main text, or Methods section.

n/a	Confirmed	
☐	☒	The exact sample size (*n*) for each experimental group/condition, given as a discrete number and unit of measurement
☒	☐	A statement on whether measurements were taken from distinct samples or whether the same sample was measured repeatedly
☐	☒	The statistical test(s) used AND whether they are one- or two-sided<br>*Only common tests should be described solely by name; describe more complex techniques in the Methods section.*
☐	☒	A description of all covariates tested
☒	☐	A description of any assumptions or corrections, such as tests of normality and adjustment for multiple comparisons
☐	☒	A full description of the statistical parameters including central tendency (e.g. means) or other basic estimates (e.g. regression coefficient) AND variation (e.g. standard deviation) or associated estimates of uncertainty (e.g. confidence intervals)
☐	☒	For null hypothesis testing, the test statistic (e.g. *F*, *t*, *r*) with confidence intervals, effect sizes, degrees of freedom and *P* value noted<br>*Give P values as exact values whenever suitable.*
☒	☐	For Bayesian analysis, information on the choice of priors and Markov chain Monte Carlo settings
☒	☐	For hierarchical and complex designs, identification of the appropriate level for tests and full reporting of outcomes
☐	☒	Estimates of effect sizes (e.g. Cohen's *d*, Pearson's *r*), indicating how they were calculated

Our web collection on statistics for biologists contains articles on many of the points above.

Software and code

Policy information about availability of computer code

Data collection	Software was used for data analyses as described below but no specific software was used to collect data.
Data analysis	The data analysis is based on open access software and documented in a dedicated github repository (https://github.com/slennart/HHA-sedaDNA).<br><br>Map (Fig. 1a): ggOceanMaps v2.0.0<br><br>Sequence processing and taxonomic classifications:<br>duplicate removal: sga 0.10.15, samtools 1.20 trimming and merging of reads: leeHom 1.2.15, taxonomic classification: bowtie2 2.4.2, damage pattern analysis I: metaDMG 0.38.0-2, damage pattern analysis II: vgan v3.0.0<br><br>Statistical analysis were performed in R v. 4.0.3:<br>pairwise Spearman rank correlation coefficients: package psych v. 2.0.12<br>interpolation of paleoenvironmental data to our sample depths: approx from package stats v. 4.0.3<br>redundancy analysis: vegan v. 2.6-8<br>radiocarbon date calibrations: rbacon v. 3.3.1 ; rice v. 1.0.0; rcarbon v. 1.5.1

For manuscripts utilizing custom algorithms or software that are central to the research but not yet described in published literature, software must be made available to editors and reviewers. We strongly encourage code deposition in a community repository (e.g. GitHub). See the Nature Portfolio guidelines for submitting code & software for further information.

Data

Policy information about availability of data

All manuscripts must include a data availability statement. This statement should provide the following information, where applicable:

- Accession codes, unique identifiers, or web links for publicly available datasets
- A description of any restrictions on data availability
- For clinical datasets or third party data, please ensure that the statement adheres to our policy

The raw sequencing data analyzed in this study have been deposited at the Sequence Read Archive (SRA) under BioProject PRJNA1211513 [https://www.ncbi.nlm.nih.gov/bioproject/PRJNA1211513].

All palaeoenvironmental datasets are open access, cited in the text, and were retrieved from PANGAEA or the supplementary information of the corresponding paper:
-MAAT: https://doi.pangaea.de/10.1594/PANGAEA.904115
-IP25: https://doi.pangaea.de/10.1594/PANGAEA.905513; https://doi.pangaea.de/10.1594/PANGAEA.911365; https://doi.org/10.17043/oden-ryder-2019-sediment-detlef-lincoln-sea-1
- Brassica- & Dinosterol: https://doi.pangaea.de/10.1594/PANGAEA.905513; https://doi.pangaea.de/10.1594/PANGAEA.911365; https://doi.org/10.17043/oden-ryder-2019-sediment-detlef-lincoln-sea-1
-Foraminifera: https://doi.pangaea.de/10.1594/PANGAEA.934100; https://doi.org/10.25921/ysgs-0037

Research involving human participants, their data, or biological material

Policy information about studies with human participants or human data. See also policy information about sex, gender (identity/presentation), and sexual orientation and race, ethnicity and racism.

Reporting on sex and gender	*Use the terms sex (biological attribute) and gender (shaped by social and cultural circumstances) carefully in order to avoid confusing both terms. Indicate if findings apply to only one sex or gender; describe whether sex and gender were considered in study design; whether sex and/or gender was determined based on self-reporting or assigned and methods used. Provide in the source data disaggregated sex and gender data, where this information has been collected, and if consent has been obtained for sharing of individual-level data; provide overall numbers in this Reporting Summary. Please state if this information has not been collected. Report sex- and gender-based analyses where performed, justify reasons for lack of sex- and gender-based analysis.*
Reporting on race, ethnicity, or other socially relevant groupings	*Please specify the socially constructed or socially relevant categorization variable(s) used in your manuscript and explain why they were used. Please note that such variables should not be used as proxies for other socially constructed/relevant variables (for example, race or ethnicity should not be used as a proxy for socioeconomic status). Provide clear definitions of the relevant terms used, how they were provided (by the participants/respondents, the researchers, or third parties), and the method(s) used to classify people into the different categories (e.g. self-report, census or administrative data, social media data, etc.) Please provide details about how you controlled for confounding variables in your analyses.*
Population characteristics	*Describe the covariate-relevant population characteristics of the human research participants (e.g. age, genotypic information, past and current diagnosis and treatment categories). If you filled out the behavioural & social sciences study design questions and have nothing to add here, write "See above."*
Recruitment	*Describe how participants were recruited. Outline any potential self-selection bias or other biases that may be present and how these are likely to impact results.*
Ethics oversight	*Identify the organization(s) that approved the study protocol.*

Note that full information on the approval of the study protocol must also be provided in the manuscript.

Field-specific reporting

Please select the one below that is the best fit for your research. If you are not sure, read the appropriate sections before making your selection.

☐ Life sciences ☐ Behavioural & social sciences ☒ Ecological, evolutionary & environmental sciences

For a reference copy of the document with all sections, see nature.com/documents/nr-reporting-summary-flat.pdf

Ecological, evolutionary & environmental sciences study design

All studies must disclose on these points even when the disclosure is negative.

Study description	In this study we investigate 12,000 years of marine ecosystem dynamics through the integration of genetic detections targeting marine mammals, micropalaeontological analysis and environmental proxy records, derived from four marine sediment cores collected around Northern Greenland.

Research sample	A total of 116 samples from the four marine sediment cores and 23 negative controls (pooled extraction blanks and library preparation blanks) were treated as samples during the whole process of sequence classification and subsequent filtering steps to monitor contamination.
Sampling strategy	We aimed at having three samples per 1,000 years and marine sediment core, however, due to the fact that the subsampling for sedaDNA was performed before the final age models were available, some time periods were only covered by 1-2 samples, while others had up to five samples per 1,000 years (Fig. 1).
Data collection	A detailed description of the laboratory workflow of sedaDNA and foraminifera samples used in this study is available in the Methods section.<br>All palaeoenvironmental datasets are open access, cited in the text, and were retrieved from PANGAEA or the supplementary information of the corresponding paper:<br>-MAAT: https://doi.pangaea.de/10.1594/PANGAEA.904115<br>-IP25: https://doi.pangaea.de/10.1594/PANGAEA.905513; https://doi.pangaea.de/10.1594/PANGAEA.911365; https://doi.org/10.17043/oden-ryder-2019-sediment-detlef-lincoln-sea-1<br>- Brassica- & Dinosterol: https://doi.pangaea.de/10.1594/PANGAEA.905513; https://doi.pangaea.de/10.1594/PANGAEA.911365; https://doi.org/10.17043/oden-ryder-2019-sediment-detlef-lincoln-sea-1<br>-Foraminifera: https://doi.pangaea.de/10.1594/PANGAEA.934100; https://doi.org/10.25921/ysgs-0037
Timing and spatial scale	The samples cover the past 12,000 years for two sediment cores (Melville Bay 26G and Lincoln Sea 12-GC) and the past 9,500 years for the other two sediment cores (Hall Basin 24PC and North-East Greenland 73G). All four sediment cores were collected on the continental shelf off the coasts of Northern Greenland. The spatial scale represented by these sediment cores is local to regional, as the sediment is influenced by oceanographic currents (e.g. West Greenland Current).
Data exclusions	We excluded all DNA sequences assigned to prokaryotes and used different filtering steps before visualizing the taxonomic detections per sediment core. The complete record of taxonomic detections after filtering is available in Data table 1 & 2. Furthermore, R scripts are provided at https://github.com/slennart/HHA-sedaDNA to guide the reader through the steps taken for arriving at the final figures.
Reproducibility	Only a part of the subsampled sediments was processed and the remainders are frozen at the Ancient DNA labs, University of Copenhagen, Denmark. To assess the reproducibility of the marine mammal occurrence data, we repeated the DNA extraction, library preparation and hybridization capture analysis for a number of samples and reported the results in Supplementary Data 9.
Randomization	During DNA extractions, we either processed all samples in the same batch (semi-automated DNA extraction), or mixed sediment samples from different depths in the respective sediment core to avoid batch effects.
Blinding	Blinding was not relevant for this study.

Did the study involve field work? ☒ Yes ☐ No

Field work, collection and transport

Field conditions	The cruise reports of ICAROS 2021 (https://data.geus.dk/pure-pdf/GEUS-R_2021_70_web.pdf), NorthGreen 2017 (https://pure-oai.bham.ac.uk/ws/portalfiles/portal/202279232/MagellanBook.pdf) & Ryder 2019 (https://doi.org/10.17043/oden-ryder-2019-expedition-5) provide detailed information on the conditions during each of the expeditions.
Location	Melville Bay 26G: 75.32° N 61.91° W, 912 m water depth<br>Hall Basin 24PC: 81.62° N 62.30° W, 520 m water depth<br>Lincoln Sea 12-GC: 82.58° N 52.53° W, 867 m water depth<br>North-East Greenland 73G: 79.07° N 11.90° W, 385 m water depth
Access & import/export	ICAROS 2021: In compliance with the Biological Diversity Convention, the Nagoya Protocol and the Greenland Parliament Act no. 3 of 3 June 2016, we obtained a Prior Informed Consent for the collection and use of genetic resources in Greenlandic waters (non-exclusive licence no. G21-041) from the Ministry of Foreign Affairs, Business, Trade and Climate of Greenland on the 10.08.2021. This licence includes an export permit for the genetic resources.<br>NorthGreen 2017: The NorthGreen17 expedition was funded by the Danish Centre for Marine Research and the Natural Science and Engineering Research Council of Canada. The project also received funding from the Danish Council for Independent Research (DFF-Sapere Aude grant no. 9064-00039B to SRI, and grants no. 7014-00113B (G-Ice project) and 0135–00165B (GreenShelf)<br>Ryder 2019: A research survey permit to enter the Northeast Greenland Nation Park, were provided by the Greenlandic/Danish authorities (Permits C-19-50 and JTHAV 2019-14122). For Canadian waters, Ryder 2019 operated under Permit IGR-940.
Disturbance	All scientific work adhered to the rules and regulations established by the Greenlandic/Danish authorities. Acoustic measurements, water column and sediment sampling was restricted to methods and areas essential to achieve the respective research goals.

Reporting for specific materials, systems and methods

We require information from authors about some types of materials, experimental systems and methods used in many studies. Here, indicate whether each material, system or method listed is relevant to your study. If you are not sure if a list item applies to your research, read the appropriate section before selecting a response.

Materials & experimental systems

n/a	Involved in the study
☒	☐ Antibodies
☒	☐ Eukaryotic cell lines
☐	☒ Palaeontology and archaeology
☒	☐ Animals and other organisms
☒	☐ Clinical data
☒	☐ Dual use research of concern
☒	☐ Plants

Methods

n/a	Involved in the study
☒	☐ ChIP-seq
☒	☐ Flow cytometry
☒	☐ MRI-based neuroimaging

Palaeontology and Archaeology

Specimen provenance

Foraminifera analyzed in this study derive from marine sediment core Melville Bay 26G (75.32° N 61.91° W, 912 m water depth) and sedimentary ancient DNA was collected from Melville Bay 26G (75.32° N 61.91° W, 912 m water depth), Hall Basin 24PC (81.62° N 62.30° W, 520 m water depth), Lincoln Sea 12-GC (82.58° N 52.53° W, 867 m water depth), and North-East Greenland 73G (79.07° N 11.90° W, 385 m water depth).

Specimen deposition

The four marine sediment cores analyzed in this study were split into two halves, of which one half was archived. The archived half of Melville Bay 26G is stored at the Geological Survey of Denmark and Greenland (GEUS) in Copenhagen, the archived half of Hall Basin 24PC and Lincoln Sea 12-GC are stored at the Department of Geological Sciences, Stockholm University, Stockholm, and the archived half of North-East Greenland 73G is stored at Department of Geoscience, Arctic Research Center, and iClimate Center, Aarhus University, Aarhus. Micropaleontological specimens (foraminifera) from Melville Bay 26G core are archived at the Geological Survey of Denmark and Greenland (GEUS) in Copenhagen.

Dating methods

The upper 10 cm of Melville Bay 26G were tested for 210Pb activity (Supplementary Data 3) using a Canberra ultralow-background Ge-detector using the Constant Rate of Supply (CRS) model110. The radiocarbon analysis was carried out using an accelerator mass spectrometer (AMS) mini carbon dating system (MICADAS) with gas targets111 on three mixed planktonic and three mixed benthic foraminifera samples (Supplementary Data 2).

☒ Tick this box to confirm that the raw and calibrated dates are available in the paper or in Supplementary Information.

Ethics oversight

No specific ethical approval was required for the analysis of foraminifera and genetic resources from marine sediments but the collection of sediments and export of genetic resources was organized in compliance with the Biological Diversity Convention, the Nagoya Protocol and Greenlandic rules and regulations (as listed above in the section "Access, import/export").

Note that full information on the approval of the study protocol must also be provided in the manuscript.

Plants

Seed stocks

Report on the source of all seed stocks or other plant material used. If applicable, state the seed stock centre and catalogue number. If plant specimens were collected from the field, describe the collection location, date and sampling procedures.

Novel plant genotypes

Describe the methods by which all novel plant genotypes were produced. This includes those generated by transgenic approaches, gene editing, chemical/radiation-based mutagenesis and hybridization. For transgenic lines, describe the transformation method, the number of independent lines analyzed and the generation upon which experiments were performed. For gene-edited lines, describe the editor used, the endogenous sequence targeted for editing, the targeting guide RNA sequence (if applicable) and how the editor was applied.

Authentication

Describe any authentication procedures for each seed stock used or novel genotype generated. Describe any experiments used to assess the effect of a mutation and, where applicable, how potential secondary effects (e.g. second site T-DNA insertions, mosiacism, off-target gene editing) were examined.
